# CD45.1/CD45.2 Congenic Markers Induce a Selective Bias for CD8^+^ T Cells during Adoptive Lymphocyte Reconstitution in Lymphocytopenia Mice

**DOI:** 10.4049/immunohorizons.2300014

**Published:** 2023-11-08

**Authors:** Rakhee Rathnam Kalari Kandy, Xiaoxuan Fan, Xuefang Cao

**Affiliations:** * Marlene and Stewart Greenebaum Comprehensive Cancer Center, University of Maryland Baltimore School of Medicine, Baltimore, MD; † Department of Microbiology and Immunology, University of Maryland Baltimore School of Medicine, Baltimore, MD

## Abstract

CD45.1/CD45.2 congenic markers have been used to track hematopoietic lineage differentiation following hematopoietic stem and progenitor cell (HSPC) transplantation. However, several studies suggest that a bias exists in CD45.1 versus CD45.2 hematopoietic cell reconstitution from HSPCs. Meanwhile, no definitive comparison has been reported for mature immune cells as to whether the CD45.1/CD45.2 disparity can skew the immune cell response. In this study, using lymphocytopenia Rag1^−/−^ CD45.2 mice as hosts, we assessed the reconstitution potential of CD45.1 versus CD45.2 lymphocytes following adoptive transfer of mature T and B cells. We have found a selective bias for CD8^+^ T cells in that CD45.1 cells showed significantly higher reconstitution compared with CD45.2 cells, whereas CD4^+^ T cells and CD19^+^ B cells showed equivalent reconstitution. These results suggest that CD45.1/CD45.2 markers may induce an alloreactive response or a survival bias specific to CD8^+^ T cells, and they therefore call for caution for using them as congenic markers in immunologic models.

## Introduction

Allelic variants of the panhematopoietic cell marker CD45, identified as CD45.1 and CD45.2, have been frequently used to track hematopoietic cells in studying the impact of genetic modification on hematopoietic reconstitution following congenic bone marrow (BM) transplants (1). However, several studies suggest that a bias exists in CD45.1 versus CD45.2 hematopoietic reconstitution. An unequal reconstitution of B cells was reported in mixed BM chimera mice that showed reduced representation of B cells of CD45.1 origin (2). Another study demonstrated that the intrinsic bias of B cell reconstitution toward CD45.2 origin is independent of an immunogenic response to the CD45.1 epitope, and it identified a sex-specific difference (1). Yet, the concern remains over alloreactivity between CD45.1 and CD45.2 epitopes. There were conflicting reports regarding whether CD45.1/CD45.2 Ags may hamper HSPC engraftment and reconstitution (3, 4). CD45.2 host-type CD8^+^TCRβ^+^ T cells were shown to hamper engraftment and reconstitution of HSPCs of CD45.1 origin, and increased intensity of conditioning the CD45.2 host significantly enhanced engraftment of CD45.1 BM cells (5). These studies mainly focused on whether CD45-disparate hosts can reject donor HSPC engraftment, thereby causing a bias in hematopoietic cell differentiation. Meanwhile, no definitive comparison has been reported for mature immune cells as to whether the CD45.1/CD45.2 markers can skew the immune cell response.

We have previously used CD45.1/CD45.2 markers to study CD4^+^ T cell proliferation after adoptive transfer into Rag1^−/−^ mice (6). To address the concern over potential bias due to CD45.1/CD45.2 disparity in mature immune cells, we tested how CD45.1/CD45.2 markers influence homeostatic proliferation after adoptive transfer into lymphocytopenia Rag1^−/−^ mice. In this study using lymphocytopenia Rag1^−/−^ CD45.2 mice as hosts, we assessed the reconstitution potential of CD45.1 versus CD45.2 lymphocytes following adoptive transfer of mature T and B cells. We have found a selective bias for CD8^+^ T cells in that CD45.1 cells showed significantly higher reconstitution compared with CD45.2 cells in the Rag1^−/−^ CD45.2 hosts. This bias is likely due to MHC class I (MHC-I)–restricted allogeneic stimulation, as CD45.1 versus CD45.2 CD4^+^ T cells and CD19^+^ B cells contained in the same graft as CD8^+^ T cells showed equivalent reconstitution.

## Materials and Methods

### Mice

Wild-type CD45.1 and CD45.2 mice, as well as Rag1^−/−^ CD45.2 mice, all in the C57BL/6J strain background, were obtained from The Jackson Laboratory and maintained in specific pathogen-free housing as previously described (6). All experiments were conducted in accordance with the animal care guidelines at the University of Maryland School of Medicine using protocols approved by the Institutional Animal Care and Use Committee.

### Adoptive transfer

Total spleen cells harvested from CD45.1 and CD45.2 mice were mixed at a 1:1 ratio. Mixed spleen cells (2–8 × 10^6^) were injected i.v. into 14 Rag1^−/−^ CD45.2 mice in three independent experiments. Both male and female mice were used. Adoptive transfer was performed between sex-matched donor and host. On day 18 after injection, spleen cells were harvested from the Rag1^−/−^ host mice for flow cytometry analyses.

### Flow cytometry

Harvested spleen cells were washed using flow buffer (PBS with 2% FBS), and Fc receptors were blocked with the addition of unlabeled anti-CD16/CD32 (BD Biosciences, 553142) for 20 min in 4°C. Cell surface markers and fixable LIVE/DEAD Fixable Aqua (Invitrogen, L34966A) were stained together in flow buffer for 30 min at 4°C and washed twice with flow buffer. Samples were run on the Aurora spectral flow cytometer (Cytek Biosciences) in the Center for Innovative Biomedical Resources at the University of Maryland School of Medicine. Unmixed samples were analyzed using FlowJo software (FlowJo).

## Results

**FIGURE 1.**
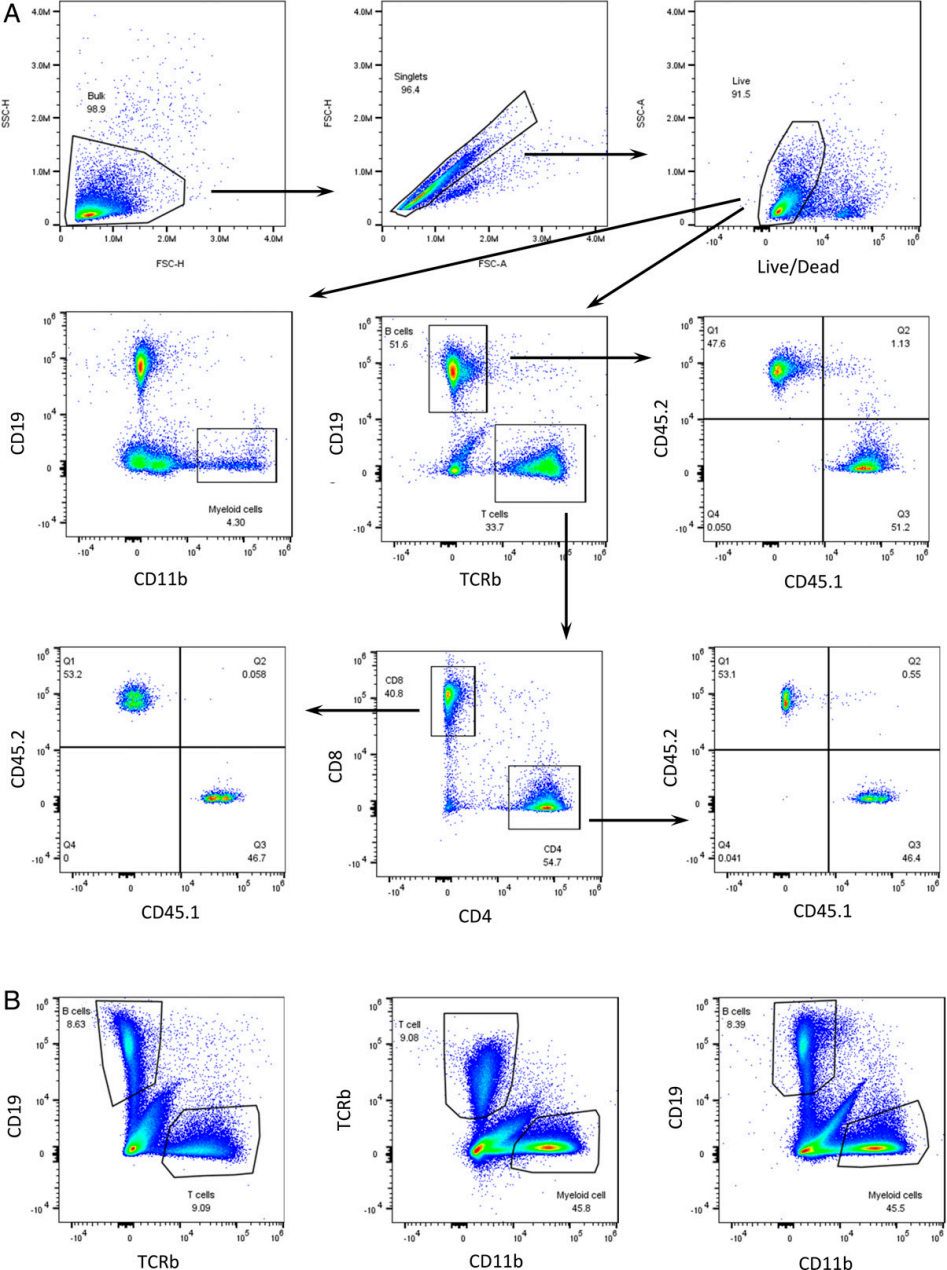
Gating scheme for analyzing the CD45.1/CD45.2 ratios in CD8^+^, CD4^+^ T cells and CD19^+^ B cells. Total spleen cells harvested from CD45.1 and CD45.2 C57BL/6J mice were mixed at a 1:1 ratio and injected i.v. into Rag1^−/−^ mice in the CD45.2 C57BL/6J strain background. (**A**) Representative flow plots showing the gating scheme for analyzing various populations in the mixed spleen cells before injection (day 0). (**B**) On day 18 after injection, spleen cells were harvested from the Rag1^−/−^ host mice. Representative flow plots show the major populations, which were analyzed for the CD45.1/CD45.2 ratios using the same gating scheme.

To test whether 45.1/CD45.2 congenic markers influence homeostatic proliferation after adoptive transfer of mature immune cells into lymphocytopenia mice, we harvested total spleen cells from CD45.1 and CD45.2 mice, mixed at a 1:1 ratio, and injected the mixed cells into Rag1^−/−^ CD45.2 mice. We performed flow cytometry analysis to determine the actual CD45.1/CD45.2 ratios in major immune cell subsets in the mixed cells before injection (day 0). All major populations in the mixed cells, including CD8^+^, CD4^+^ T cells, CD19^+^ B cells, and CD11b^+^ myeloid cells, showed an actual ratio of ∼1 (Fig. 1A), indicating that there is no significant difference in the frequencies of these subsets between CD45.1 and CD45.2 donor mice. Based on our previous study of time course with T cell proliferation in this model (6), we chose an endpoint of day 18 after adoptive transfer, a time point for which we have observed significant T cell reconstitution. Then, we harvested total spleen cells from the Rag1^−/−^ host mice and performed flow cytometry to analyze the frequencies of these major populations and the CD45.1/CD45.2 ratios among them. As there are no endogenous T or B cells in the Rag1^−/−^ host mice, adoptive transfer indeed led to significant reconstitution of donor-derived T and B cells (Fig. 1B), although the dominant population in the Rag1^−/−^ host was still the endogenous CD11b^+^ myeloid cells.

**FIGURE 2.**
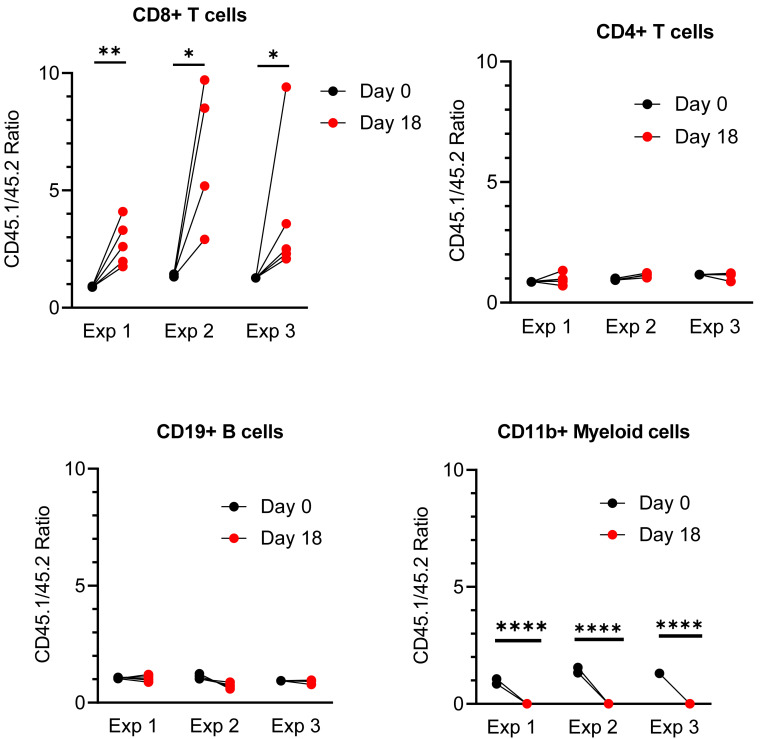
Significantly increased reconstitution in CD45.1 versus CD45.2 in CD8^+^ T cells, but not in CD4^+^ T cells or CD19^+^ B cells. Results were summarized from three independent experiments; *n* = 4 or 5 Rag1^−/−^ host mice for each experiment. Mixed spleen cells (2–8 × 10^6^) were injected into each Rag1^−/−^ host mouse. Unpaired *t* tests were performed to compare the CD45.1/CD45.2 ratios in the major populations of spleen cells between day 0 preinjection and day 18 postinjection. *p < 0.05, **p < 0.01, ****p < 0.0001.

When we gated on each of these major populations to analyze the CD45.1/CD45.2 ratio, we found that the ratio in CD8^+^TCRβ^+^ T cells significantly increased to a range of 2–10 in a total of 14 Rag1^−/−^ host mice used in three independent experiments (Fig. 2). In contrast, the CD45.1/CD45.2 ratios in CD4^+^TCRβ^+^ T cells and CD19^+^ B cells remained unchanged at ∼1. In contrast, this ratio went down to 0 in CD11b^+^ myeloid cells, suggesting that donor-type CD45.1 CD11b^+^ myeloid cells did not expand or survive to a detectable level due to the dominance of host-type CD45.2 CD11b^+^ myeloid cells. Nevertheless, the disappearance of donor-type CD11b^+^ cells, dramatic yet expected, serves as a negative control for the significant reconstitution of donor-derived T and B cells in this lymphocytopenia model. Of note, to address the concern over potential sex-related bias, adoptive transfer was performed between sex-matched donor and host. Both male and female mice were used in these experiments, which showed equivalent phenotypes.

## Discussion

In summary of the results from this lymphocytopenia Rag1^−/−^ model that expresses CD45.2, we conclude that both CD45.1 and CD45.2 donor T and B cells undergo significant reconstitution. Notably, CD45.1 CD8^+^ T cells undergo significantly higher reconstitution, likely resulting from homeostatic proliferation plus allogeneic stimulation by the CD45-diparate Ag. That CD4^+^ T cells and CD19^+^ B cells do not show a bias suggests that the CD45-disparate epitope is highly likely serving as an MHC-I–restricted minor histocompatibility Ag. However, no significant increase in absolute cell number was observed for donor-derived CD8^+^ T cells from the initial input when absolute cell numbers were calculated, suggesting that the observed T cell reconstitution may be a collective result from proliferation, death, and survival. Alternatively, a recent study indicated that the CD45.1 mice have a point mutation in the Ncr1 locus that leads to an increased innate IFN-γ response to viral infection (7). Such a cell-intrinsic bias could drive CD45.1^+^CD8^+^ T cells toward better expansion or survival. Furthermore, earlier studies suggested that CD45 may function as a costimulatory molecule for T cell activation and signaling (8, 9), raising the possibility that CD45.1 may function better than CD45.2 in promoting T cell proliferation. However, that CD45.1 and CD45.2 CD4^+^ T cells show equivalent reconstitution suggests that their costimulatory functions are equivalent in CD4^+^ T cells in this model.

Beyond the concern for alloreactivity, several studies with competitive BM transplants reported functional differences in the relative homing efficiency and hematopoietic reconstitution potential between CD45.1 and CD45.2 HSPCs, attributed presumably to a congenic interval that differs for 42 Mb encoding ∼300 genes (10, 11). However, a genetically intrinsic impact on homing and reconstitution efficiency resulting from this cluster of genes is unlikely to be the cause of the selective bias for CD8^+^ T cells in our model, because CD4^+^ T cells and CD19^+^ B cells showed equivalent reconstitution. In contrast to HSPCs, our data suggest that in mature immune cell subsets, the CD45.1 versus the CD45.2-disparate epitope may induce an MHC-I–restricted mild alloreactive response causing higher expansion or survival of CD45.1 CD8^+^ T cells. It is less likely that genetically intrinsic factors embedded in the CD45 interval skew mature immune cell proliferation, which, if real, should have broader impact on all mature immune cell subsets, rather than a selective bias on CD8^+^ T cells alone.
